# The complement system is activated in synovial fluid from subjects with knee injury and from patients with osteoarthritis

**DOI:** 10.1186/s13075-016-1123-x

**Published:** 2016-10-06

**Authors:** André Struglics, Marcin Okroj, Per Swärd, Richard Frobell, Tore Saxne, L. Stefan Lohmander, Anna M. Blom

**Affiliations:** 1 Department of Clinical Sciences Lund, Orthopaedics, Lund University, Faculty of Medicine, BMC C12, SE-221 84 Lund, Sweden; 2 Department of Translational Medicine, Division of Medical Protein Chemistry, Lund University, Faculty of Medicine, Lund, Sweden; 3 Department of Medical Biotechnology, Intercollegiate Faculty of Biotechnology UG-MUG, Medical University of Gdańsk, Gdańsk, Poland; 4 Department of Clinical Sciences Lund, Rheumatology, Lund University, Faculty of Medicine, Lund, Sweden

**Keywords:** Complement, Knee injury, Osteoarthritis, Synovial fluid

## Abstract

**Background:**

The complement system is suggested to be involved in the pathogenesis of osteoarthritis (OA), and proinflammatory cytokines may play a role in OA development by inducing proteases. The association between complement factors, cytokines and OA has not been investigated. The aim of the present study was to explore the involvement of the complement system after knee trauma and in OA.

**Methods:**

C4d, C3bBbP and soluble terminal complement complex (sTCC) resulting from complement activation were immunoassayed in synovial fluid from subjects with healthy knees (reference), OA, rheumatoid arthritis (RA; positive control), pyrophosphate arthritis (PPA; positive control) and knee injury; other biomarkers were previously assessed. Magnetic resonance imaging was used to assess joint injuries.

**Results:**

Compared with levels in the reference group, the median concentrations of C4d, C3bBbP and sTCC in the OA, RA, PPA and knee injury groups were 2- to 34-fold increased (*p* < 0.001 to *p* = 0.044). For the knee injury group, the median concentrations of C4d, C3bBbP and sTCC were 5- to 12-fold increased (*p* < 0.001) at the day of injury; after 3–12 weeks, C3bBbP and sTCC concentrations were similar to reference levels; and C4d was still increased several years after injury. In the 0–12 weeks period after injury, the concentrations of C4d, C3bBbP and sTCC correlated positively with levels of interleukin (IL)-1β, IL-6 and tumour necrosis factor α (*r*_s_ range 0.232–0.547); none of the measured complement factors correlated with proteolytic fragments of aggrecan or cartilage oligomeric matrix protein. Knees with osteochondral fracture, with or without disrupted cortical bone, had higher concentrations of C4d (*p* = 0.014, *p* = 0.004) and sTCC (*p* = 0.004, *p* < 0.001) compared with knees without fractures.

**Conclusions:**

The complement system is activated in OA and after knee injury. Following knee injury, this activation is instant and associated with inflammation as well as with the presence of osteochondral fractures.

**Electronic supplementary material:**

The online version of this article (doi:10.1186/s13075-016-1123-x) contains supplementary material, which is available to authorized users.

## Background

The complement system as a part of innate immunity is one of the first lines of defence against invading pathogens. Apart from this basic, evolutionarily conserved function, it also plays a role in scavenging of cellular debris and immune complexes, opsonisation and guidance of adaptive immunity by anaphylatoxins and stimulation of B and T lymphocytes [1]. Importantly, complement is an aggressive system capable of targeting even its own cells and tissues when improperly regulated by endogenous inhibitors and/or autoantibodies, or because of gain-of-function mutations in complement components [1, 2]. Such autoreactivity was shown to be an important constituent of pathological mechanisms of inflammatory and autoimmune diseases, including rheumatoid arthritis (RA) [3]. Cartilage degradation products released in the course of joint degenerative diseases form a separate class of potent complement modulators [4–7].

Joint injury is a major risk factor for the development of post-traumatic osteoarthritis (OA) [8]. Mechanical injury to the joint leads to haemarthrosis, death of chondrocytes and bone cells [9, 10], as well as damage to the tissue extracellular matrix (ECM) directly or mediated by chondrocytes and synoviocytes via increased expression and activation of matrix-degrading enzymes stimulated by proinflammatory cytokines [11]. Furthermore, complement activation has been implicated in the connective tissue repair and inflammatory response initiated by trauma [12, 13]. These observations led us to hypothesize that mechanical joint injury may be an initial trigger of local inflammation, including activation of complement, and that subsequent ECM erosion of injured joints may reinforce complement activation in a positive feedback manner. Monitoring of the complement activation profile after injury could give information potentially important for the design of therapeutic strategies.

**Fig. 1:**
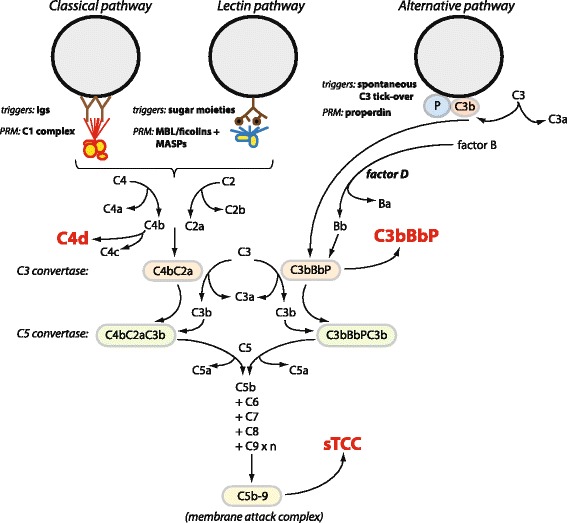
A scheme of the complement system. Complement can be activated via three different routes. The classical and lectin pathways have their own specific pattern recognition molecules (PRMs), whereas the alternative pathway is activated by a spontaneous tick-over of C3 and deposition of C3b molecules onto permissive surfaces, which is facilitated by properdin (P). All pathways converge at the stage of C3 convertases, which catalyse breakdown of C3 into C3a and C3b molecules. When C3b molecules bind to the convertase complex, it gives rise to the C5 convertase. Except for the triggers indicated for each pathway in the scheme, apoptotic and necrotic cellular debris as well as degradative protein fragments from the extracellular matrix can also trigger all three pathways [4, 32]. In *red* and *boldface type* are the markers of complement activation analysed in our study: C4d, the end degradation product of C4b; C3bBbP, a soluble form of alternative C3 convertase including properdin; and sTCC, soluble terminal complement complex (a soluble form of C5b-9). *Factor D* Serine protease that cleaves factor B, *Igs* Immunoglobulins, *MASP* Mannose-binding lectin-associated serine protease, *MBL* Mannose-binding lectin

We therefore investigated complement activation in OA and the time-dependent activation after knee injuries. RA and pyrophosphate arthritis (PPA) samples were used as positive controls for complement activation. We measured complement activation products characteristic of classical and/or lectin (C4d) and alternative (C3bBP) pathways, as well as the soluble terminal complement complex (sTCC), in the synovial fluid from different patient diagnostic groups (Fig. 1).

## Methods

### Subjects

In our cross-sectional convenience cohort, 294 patients with knee injury had synovial fluid aspirated once from their knees without lavage; of those, 112 subjects did not fulfil the inclusion criteria of a randomized controlled trial [14], and the remaining 182 subjects had been studied in previous cross-sectional investigations [15–18] (Table 1). On the basis of time between injury and synovial fluid aspiration, the subjects with knee injury were stratified into a recent injury group (sampling 0–83 days, or 0–12 weeks, after injury) and an old injury group (sampling 1–37 years after injury), and these groups were substratified by time after injury into groups of 24–46 subjects each (Table 1). From another convenience cohort, we used 23 reference subjects (without a history of knee symptoms or knee injury, or with normal findings in clinical, radiographic and arthroscopic examinations), and 24 patients with OA, 32 with RA and 25 with PPA (RA and PPA, positive controls for complement activation) who had synovial fluid aspirated from their knees (Table 1). These samples have been used in previous investigations [14–19]. For the patients in the OA and old injury groups, an OA score (ranging from 1 to 10) for their affected joints was assessed by arthroscopy and radiography, with a score of 1 representing a normal joint by arthroscopy and radiography, scores of 2–5 representing an increasing extent and severity of fibrillation and clefts by arthroscopy and appearing normal on a radiograph, and scores of 6–10 representing increasing degrees of radiographic joint space narrowing [20].

**Table 1:** Characteristics of the study subjects

Main diagnostic groups	Subgroups of injury	Duration, range (median)	*n* (% women)	Age in years, median (range)	Differences in age, *p* value
Reference subjects	–	NA	23 (22)	26 (15–58)	–
Osteoarthritis^a^	–	0–14.8 years (2.7)	24 (42)	64 (36–86)	**<0.001**
Rheumatoid arthritis^b^	–	0–43.0 years (10)	32 (63)	64 (32–83)	**<0.001**
Pyrophosphate arthritis	–	0–9.8 years (0.01)	25 (40)	75 (41–92)	**<0.001**
Knee injury^c^	–	0–36.9 years (0.02)	294 (27)	29 (13–65)	0.842
	Recent injury	0–83 days (4)	219 (25)	26 (13–64)	0.705
	Recent injury, substratification	0 days (0)	35 (23)	30 (14–47)	0.991
		1 day (1)	39 (21)	24 (13–57)	0.211
		2–3 days (3)	31 (29)	26 (16–54)	0.914
		4–7 days (5)	46 (28)	25 (14–64)	0.492
		8–22 days (11)	44 (30)	23 (14–57)	0.151
		23–83 days (32)	24 (13)	38 (21–59)	**0.033**
	Old injury^d^	1–36.9 years (3.3)	75 (33)	32 (18–65)	0.090
	Old injury, substratification	1–3 years (2.0)	35 (40)	33 (18–61)	0.149
		3.01–36.9 years (5.1)	40 (28)	30 (18–65)	0.121

The injury groups are presented with stratification based on time between injury and synovial fluid aspiration. Differences in age between patients and reference groups were analysed using Student’s *t* test, and significant values (*p* < 0.05) are indicated by boldface type. *Duration* refers to time between injury or onset of disease and sampling. *NA* Not applicable

^a^Symptomatic and/or radiographically diagnosed idiopathic osteoarthritis (with no history of knee trauma), with information of disease duration from 17 of 24 patients. The OA score for the OA group was as follows: median = 7, range = 3–9 (information from 15 of 24 patients)

^b^Rheumatoid arthritis, with information of disease duration from 29 of 32 patients, taking the following medications: no information (*n* = 1), no medication (*n* = 3), non-steroidal anti-inflammatory and/or disease-modifying drugs (*n* = 28)

^c^The clinical diagnosis of the subjects with knee injury was as follows: isolated anterior cruciate ligament (ACL) or posterior cruciate ligament (PCL) injuries (ACL *n* = 39, PCL *n* = 4), ACL injury with meniscal tear (*n* = 69), ACL injury with meniscal tear and other ligament injuries (*n* = 37), ACL injury with other ligament injuries (*n* = 47), isolated meniscal tear (*n* = 58), meniscal tear with other (not ACL) ligament injuries (*n* = 7), patellar dislocation with or without soft tissue injuries (*n* = 13), other types of injuries (medial or lateral collateral ligament tears *n* = 7, give-way *n* = 2), no signs of soft tissue injury (*n* = 10)

^d^Six patients in the old injury group had post-traumatic OA (based on OA score ≥5 [20]), and the group had the following OA score: median = 2, range = 1–8 (information from 63 of 75 patients)

Synovial fluid was aspirated (without lavage) from each subject at one time point only, centrifuged at 3000 × *g* for 10 minutes at 4 °C, and the supernatants were then stored at −80 °C.

### Analysis of C4d, C3bBbP and sTCC in synovial fluid

Concentrations of C4d [21], C3bBbP and sTCC (C5b-9) [22] in synovial fluid were measured by performing sandwich enzyme-linked immunosorbent assays (ELISAs). Briefly, for the C4d assay, a capture antibody against a C4d neoepitope, together with a mouse anti-C4d detection antibody (A253; Quidel, San Diego, CA, USA), followed by peroxidase-conjugated goat anti-mouse (P0447; Dako, Carpinteria, CA, USA) were used. For the C3bBbP assay, a capture antibody against properdin (A235; Quidel), together with a rabbit anti-C3c detection antibody (P0062; Dako), followed by peroxidase-conjugated goat anti-rabbit (P0448; Dako) were used. For the sTCC assay, we used a monoclonal capture antibody against a C9 neoepitope (clone ae11, HM2167; Hycult Biotech, Uden, the Netherlands), together with an in-house biotinylated monoclonal anti-C6 antibody (A219; Quidel), followed by a streptavidin-HRP reagent. The read-out of each of these assays was given in complement activation units (CAU), a defined arbitrary unit set for the International Complement Standard #2 sample, which is serum pooled from approximately 1000 healthy individuals and incubated with activators of all three complement pathways [22].

### Other biomarkers and cytokines

A subset of synovial fluid samples from the recent injury group (*n* = 111–181) were previously assayed for the following biomarkers: sulfated glycosaminoglycan (sGAG, a marker for total aggrecan); ARGS neoepitope of aggrecan (ARGS-aggrecan from aggrecanase cleavage at the TEGE^392^↓^393^ARGS site of aggrecan); osteocalcin, secreted protein acidic and rich in cysteine (SPARC), also known as osteonectin, osteopontin, cartilage oligomeric matrix protein (COMP); type II collagen epitope (C2C); osteopontin; and the proinflammatory cytokines interleukin (IL)-1β, IL-6, IL-8 and tumour necrosis factor α (TNF-α) [14, 17, 19, 23].

### Image acquisition and analysis

Magnetic resonance imaging (MRI) using a 1.5-Tesla scanner was conducted in a subfraction of the recent injury group as described elsewhere [19]. Briefly, synovial fluid was aspirated 0–23 days after injury, and MRI scans were acquired within a median of 8 days (range 1–38) after injury in 98 of the 219 subjects of the recent injury group. MRI studies were assessed by an experienced musculoskeletal radiologist for anterior cruciate ligament (ACL) tears, meniscal tears, post-traumatic bone marrow lesions (BMLs), apparent cartilage defects (evident irregularity of cartilage) and osteochondral (OC) fractures with or without disrupted cortical bone. Such fractures were considered a proxy for the strength of impact forces applied over the articular surface at the time of injury [19, 24]. These factors were classified as present or absent for the entire knee.

### Statistics and calculations

According to Shapiro-Wilk tests, synovial fluid C4d, C3bBbP and sTCC concentrations were not normally distributed in all diagnostic groups; therefore, when all groups were compared, non-parametric analysis was conducted. Between-groups comparisons were made using Mann-Whitney rank-sum tests, and for correlation analysis, Spearman’s rank correlation (*r*_s_) analysis was used. Student’s *t* test was used for comparison of age between subject groups. All of these tests were two-tailed. For a subset (*n* = 98) of the recent injury group, one-way analysis of covariance (ANCOVA) was used to investigate differences in synovial fluid C4d, C3bBbP and sTCC concentrations with regard to structural joint damage as visualized by MRI. After log_10_ transformation, the data were normally distributed according to the Shapiro-Wilks test, and ANCOVA was done with adjustments for days between injury and synovial fluid aspiration, age and sex. The mean and 95 % CI were calculated using log_10_-transformed data, but they are presented as linear data.

Samples with concentrations below the lower limit of detection (LLOD) were imputed and given a value equal to half the value of the LLOD. The synovial fluid concentrations of C4d, C3bBbP and sTCC, as well as the amount of samples with imputed values (between 0 and 17 % depending on groups) in the different subject groups, are provided in Additional file: Table S1. In the group-level analysis, the imputed values were included. In the correlation analysis, samples with and without (measured values only) imputed values were analysed, but significance was considered only when both analyses showed correlation, and the presented data (*r*_s_ and *p* values) include imputed values. IBM SPSS version 21 software (IBM, Armonk, NY, USA) was used for statistical analysis, and *p* values less than 0.05 were considered significant. Expressions such as “higher” and “increase” in the text are based on statistically significant differences.

## Results

### Technical performance of the C4d, C3bBbP and sTCC ELISAs with synovial fluid

The LLOD and upper limit of detection (ULOD) for the C4d ELISA were 0.05 and 25 CAU, respectively (Additional file 2: Table S2). Within this range, good dilution linearity was observed for synovial fluid control samples diluted 1:5 to 1:40, mean recoveries between 97 % and 104 %, and reference and patient synovial fluid samples were used at the same dilutions. The LLOD and ULOD for the C3bBbP and sTCC assays were 0.05 and 50, respectively, and 0.02 and 10 CAU, respectively (Additional file 2: Table S2). The control synovial fluid samples showed poor dilution linearity in the C3bBbP and sTCC assays, mean recoveries were 54–150 % for C3bBbP and 60–141 % for sTCC; therefore, all analysis of the reference and patient synovial fluid samples were done at the same dilution of 1:20. Spiking the synovial fluid control samples with different amounts of standards showed good recovery for the C4d and C3bBbP assays (mean recoveries between 79 % and 99 % and between 96 % and 107 %, respectively), while the sTCC assay showed lower recovery of 68–77 %. As also shown for measurements in serum and plasma samples [21, 22], the C4d, C3bBbP and sTCC concentrations in synovial fluid were not affected by repeated freeze-thaw cycles, tested for up to 15 cycles (Additional file 2: Table S2). The intra-assay coefficient of variation (CV, within plates) for the synovial fluid control samples was approximately similar between the C4d, C3bBbP and sTCC assays (between 9 % and 10 %), while the inter-assay CV (between plates) was higher for the C3bBbP and sTCC assays (both 23 %) compared with the C4d assay (16 %) (Additional file 2: Table S2).

### Concentrations of C4d, C3bBbP and sTCC in synovial fluid were higher in the arthritis groups than in the reference group

**Fig. 2:**
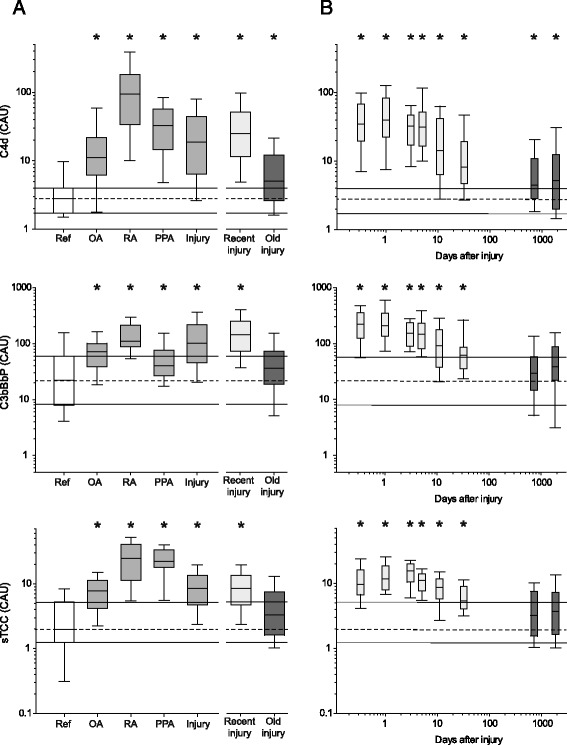
Synovial fluid concentrations of C4d, C3bBbP and soluble terminal complement complex (sTCC). **a** Box plots with subjects ordered by the diagnostic groups: reference, osteoarthritis (OA), rheumatoid arthritis (RA), pyrophosphate arthritis (PPA), and knee injuries split into recent injury and old injury. **b** Knee injury samples ordered by days after injury in subgroups of 24–46 subjects (Table 1), showing recent injury and old injury groups. Boxes show the quartiles (median, 25th and 75th percentiles) with error bars and whiskers for the 10th and 90th percentiles. The quartiles of the reference group are extended as *thin horizontal lines* in both panels for comparison. Statistically significant group differences, determined by Mann-Whitney *U* test, versus the reference group are indicated by asterisks (*) and are presented in Additional file 1: Table S1. *CAU* Complement activation units

The concentrations of C4d, C3bBbP and sTCC were higher in the OA, RA and PPA groups than in the reference group. Median levels increased between 4- and 34-fold (C4d), 2- and 5-fold (C3bBbP), and 4- and 12-fold (sTCC) (Fig. 2a, Additional file 1: Table S1). The highest synovial fluid concentrations of C4d, C3bBbP and sTCC were found in the RA group (Fig. 2a, Additional file 1: Table S1).

The synovial fluid concentrations of C4d, C3bBbP and sTCC did not differ between men and women in the different subject groups (Additional file 3: Table S3). The subjects in the OA, RA and PPA groups were older than the subjects in the reference group (Table 1). Also, there was a positive correlation in the OA and PPA groups between age and concentrations of sTCC, and in the reference group for C4d; no other correlation between age and concentration of complement products was found in the subject groups (Additional file 3: Table S3). On one hand, this suggests that, compared with the reference group, the increased levels of C3bBbP in the OA, RA and PPA groups, as well as the increased levels of sTCC in the RA group, were due to disease mechanisms and not to differences in age. On the other hand, the increased levels of C4d in the OA, RA and PPA groups, as well as the increased levels of sTCC in the OA and PPA groups, could also be due partially to differences in age between the disease and reference groups.

### Concentrations of C4d, C3bBbP and sTCC in synovial fluid were higher in the knee injury group than in the reference group

The concentrations of C4d, C3bBbP and sTCC were higher in the knee injury group than in the reference group, with median increases of 7-, 5- and 4-fold, respectively (Fig. 2a, Additional file 1: Table S1). The synovial fluid concentrations of C4d, C3bBbP and sTCC did not differ between men and women in the knee injury group (Additional file 3: Table S3). There was no difference in mean age between the reference group and the knee injury group (0–37 years after injury), or between the reference group and the recent injury (0–83 days after injury) and old injury (1–37 years after injury) subgroups (Table 1). This suggests that the increased levels of activated complement products in the synovial fluid of injured knees compared with reference knees were due to the knee trauma.

The elevated concentrations of C4d, C3bBbP and sTCC were seen immediately after injury (day 0, the day of injury) as 12-, 10- and 12-fold increases of median levels compared with the reference group, with a lower still significantly elevated level in knees aspirated over the first 12 weeks after injury (Fig. 2b, Additional file 1: Table S1). As seen by using correlation analysis, the synovial fluid concentrations of C4d, C3bBbP and sTCC in the recent injury group (0–83 days after injury) decreased with time after injury (Fig. 2b, Additional file 3: Table S3), while no lower levels were observed over later times in the old injury group (1–37 years after injury) (Additional file 3: Table S3). Many years after injury, the synovial fluid concentration of C4d was still elevated 2-fold compared with levels in the reference group (Fig. 2b, Additional file 1: Table S1), although this increase could be due in part to the fact that some patients in the old injury group had post-traumatic OA (Table 1).

### Correlation between activated complement products

In all the subject groups, the synovial fluid concentrations of C4d and sTCC showed a moderate to strong positive correlation (Table 2). In the reference, RA and knee injury groups, there was a moderate to strong positive correlation between C4d and C3bBbP, and in the RA and knee injury groups, there was also a moderate positive correlation between C3bBbP and sTCC (Table 2).

**Table 2:** Correlation between C4d, C3bBbP and sTCC in reference and patient groups

	Reference (*n* = 23)		Osteoarthritis (*n* = 24)		Rheumatoid arthritis (*n* = 32)	
	C4d	C3bBbP	C4d	C3bBbP	C4d	C3bBbP
C3bBbP	**0.428 (0.041)**	–	0.080 (0.710)	–	**0.536 (0.002)**	–
sTCC	**0.625 (0.001)**	0.409 (0.053)	**0.754 (<0.001)**	–0.298 (0.157)	**0.752 (<0.001)**	**0.509 (0.003)**
	Pyrophosphate arthritis (*n* = 25)		Knee injury (*n* = 294)			
	C4d	C3bBbP	C4d	C3bBbP		
C3bBbP	–0.085 (0.685)	–	**0.674 (<0.001)**	–		
sTCC	**0.496 (0.012)**	0.345 (0.091)	**0.643 (<0.001)**	**0.494 (<0.001)**		

*sTCC* Soluble terminal complement complex

Correlation using Spearman’s rho (*r*_s_) between synovial fluid biomarkers of complement system analysed in different subject groups. Significant correlations (*p* < 0.05) are indicated by boldface type. The table shows *r*_s_ with *p* values in brackets

### Correlation between activated complement products and other biomarkers in the recent injury group

For the recent injury group (0–83 days after injury), we performed a correlation analysis between synovial fluid concentrations of the activated complement factors and the levels of proinflammatory cytokines and biomarkers of cartilage (sGAG, ARGS-aggrecan, COMP and C2C) and bone (osteocalcin, SPARC and osteonectin). A weak to moderate positive correlation was found for the C4d, C3bBbP and sTCC concentrations and the levels of cytokines, where C4d and TNF showed the strongest correlation (*r*_s_ = 0.547). No correlation was found between the concentrations of activated complement products and the levels of aggrecan markers sGAG and ARGS-aggrecan or between activated complement products and COMP (Table 3). A weak negative correlation was found between the concentrations of type II collagen epitope C2C and the level of C4d. Also, C4d, C3bBbP and sTCC concentrations showed weak to moderate positive correlations with the levels of SPARC, and concentrations of C4d and C3bBbP had a weak positive correlation with the levels of osteocalcin (Table 3).

**Table 3:** Correlation between C4d, C3bBbP, sTCC and other biomarkers in the recent injury group

Biomarkers	*n*	C4d	C3bBbP	sTCC
IL-1β	112	**0.397 (<0.001)**	**0.232 (0.014)**	**0.297 (0.001)**
IL-6	112	**0.364 (<0.001)**	**0.235 (0.013)**	**0.268 (0.004)**
IL-8	112	**0.355 (<0.001)**	0.183 (0.054)	**0.372 (<0.001)**
TNF-α	112	**0.547 (<0.001)**	**0.317 (0.001)**	**0.391 (<0.001)**
sGAG	181	0.108 (0.148)	0.092 (0.218)	0.132 (0.077)
ARGS	111	−0.023 (0.811)	−0.113 (0.238)	0.176 (0.065)
COMP	112	−0.019 (0.845)	−0.149 (0.116)	−0.057(0.549)
C2C	164	**−0.181 (0.020)**	−0.101 (0.197)	−0.085 (0.279)
Osteocalcin	112	**0.265 (0.005)**	**0.192 (0.043)**	0.016 (0.871)
SPARC	112	**0.476 (<0.001)**	**0.221 (0.019)**	**0.401 (<0.001)**
Osteopontin	112	0.167 (0.079)	0.098 (0.302)	0.165 (0.083)

*Abbreviations: IL* Interleukin, *TNF-α* Tumour necrosis factor α, *sGAG* Sulfated glycosaminoglycan, *ARGS* ARGS-aggrecan, *COMP* Cartilage oligomeric matrix protein, *C2C* Type II collagen epitope, *SPARC* Secreted protein acidic and rich in cysteine, *sTCC* Soluble terminal complement complex

Correlation using Spearman rho (*r*_s_), between synovial fluid biomarkers was analysed in samples from the recent injury group (0–83 days after injury). Significant correlations (*p* < 0.05) are shown in boldface type. The table shows *r*_s_ with *p* values in brackets. References to biomarkers: cytokines, ARGS, sGAG, osteocalcin, SPARC, osteopontin [14], COMP [19], C2C [23]

### Association between activated complement products and MRI findings in the recent injury group

For a subset of the recent injury group, we compared MRI features with synovial fluid concentrations of the complement factors. Knees with any OC fracture (with or without disrupted cortical bone) had higher concentrations of C4d and sTCC than knees without an OC fracture (Table 4). Knees with an OC fracture with disrupted cortical bone had higher concentrations of C4d and sTCC than knees without an OC fracture, and they also higher concentrations of C4d than knees with an OC fracture without disrupted cortical bone (Table 4). There were no significant differences between knees with an OC fracture without disrupted cortical bone and knees without OC fracture (data not shown). There was no difference in the synovial fluid concentrations of the complement factors between knees that acquired an ACL injury and knees without an ACL injury, although knees with a meniscal tear had lower concentrations of sTCC than knees without a meniscal tear (Table 4).

**Table 4:** C4d, C3bBbP, sTCC concentrations in the recent injury group in relation to structural features

	Any OC fracture (*n* = 67)	vs.	No OC fracture (*n* = 31)	vs.	OC fracture with disrupted cortical bone (*n* = 38)	vs.	OC fracture without disrupted cortical bone (*n* = 29)
	Mean (95 % CI)	*p* value	Mean (95 % CI)	*p* value	Mean (95 % CI)	*p* value	Mean (95 % CI)
C4d	38.90 (32.01–47.29)	**0.014**	23.92 (15.17–37.70)	**0.004**	45.42 (33.95–60.79)	**0.025**	31.76 (25.00–40.35)
C3bBbP	235.61 (203.19–273.15)	0.478	213.60 (162.33–281.06)	0.612	237.30 (195.79–287.61)	0.637	233.35 (182.64–298.19)
sTCC	12.81 (11.35–14.47)	**0.004**	9.27 (7.20–11.94)	**<0.001**	14.26 (12.20–16.68)	0.056	11.13 (9.20–13.46)
	ACL injury (*n* = 60)	vs.	No ACL injury (*n* = 38)				
	Mean (95 % CI)	*p* value	Mean (95 % CI)				
C4d	34.34 (28.39–41.52)	0.418	31.86 (20.87–48.66)				
C3bBbP	231.42 (198.93–269.28)	0.417	223.67 (174.62–286.48)				
sTCC	11.53 (10.26–12.97)	0.885	11.61 (9.08–14.85)				
	Meniscal tear (*n* = 43)	vs.	No meniscal tear (*n* = 55)				
	Mean (95 % CI)	*p* value	Mean (95 % CI)				
C4d	29.36 (21.57–39.98)	0.565	36.86 (28.42–47.80)				
C3bBbP	204.27 (172.78–241.49)	0.399	249.23 (205.21–302.69)				
sTCC	9.91 (8.46–11.62)	**0.013**	13.05 (11.08–15.37)				

*Abbreviations: ACL* Anterior cruciate ligament, *OC* Osteochondral, *sTCC* Soluble terminal complement complex

Magnetic resonance imaging results were available for a subfraction of the recent injury group (*n* = 98). Between-groups statistical testing was performed using analysis of covariance of log_10_-transformed concentrations with adjustment for days between the injury and synovial fluid aspiration, age at injury, and sex. Significant differences (*p* < 0.05) are shown in boldface type. Concentrations in complement activation units are presented as linear data as means with 95 % CI in brackets

## Discussion

Using synovial fluid from subjects in different diagnostic groups, we show that C4d, C3bBbP and sTCC concentrations were elevated not only in our positive controls RA and PPA but also in OA knees and in knee injury compared with levels in the healthy reference group knees. In injured knees, this increase was immediate, seen in knees aspirated on the day of injury, compared with lower levels in knees aspirated up to weeks after injury. Compared with levels of reference knees, knees aspirated several years after injury only showed elevated levels of C4d.

Complement C4d fragment (a proteolytic fragment of C4 with unknown biological function) is an early-stage marker for the classical and lectin pathways, C3bBbP (a C3 convertase) is an early stage marker for the alternative pathway, and sTCC (also known as *membrane attack complex* [MAC]) is a late-stage marker for all three pathways [1]. In autoimmune diseases such as RA, all three pathways are activated [3]. Analysis of synovial fluid by mass spectrometry and ELISA has shown that levels of complement components are elevated in patients with OA compared with levels found in healthy individuals [25–27]. Further, patients with OA had increased synovial fluid concentrations of C3a, an anaphylatoxin (i.e., stimulator of inflammation) that can be generated from all three pathways, and of sTCC compared with levels in healthy control subjects [27]. Our results confirm that patients with OA have increased concentrations of sTCC, and they extend previous findings by showing that the alternative pathway, together with the classical and/or lectin complement pathways, was activated in the synovial fluid of patients with OA and after knee injury.

Complement factors present in the synovial fluid after knee trauma may originate from intra-articular bleeding (i.e., haemarthrosis from the ligaments and/or from the synovium) or may be produced by synovial cells (leucocytes and synoviocytes) and/or chondrocytes [27–31]. Joint bleeding is very common after acute knee injury, and 96 of the 98 recent injury samples with available MRI data had haemarthrosis in our study. At the moment of knee trauma, impact forces can also result in BMLs and OC fractures visualized by MRI. On the basis of previous evidence [24], we anticipated that the presence of OC fractures could be used as a proxy for a strong impact trauma and that disruption of the cortical bone may be an indicator of more severe joint trauma. Complement is involved in fracture healing, where it affects osteoblasts and osteoclasts. These bone cells also produce C3 and C5, and type I collagen is capable of activating platelets, forming sTCC and upregulating receptors for C3a and C5a [32, 33]. At the fracture edge of matrix cracks, the chondrocyte death rate is very high, and damage-associated molecular patterns released from dying cells (i.e., cellular debris such as mitochondria, histones, DNA) contributes to an activation of the innate system [10, 32, 34] and directly triggers C1q [35]. Further, factor VII-acting serine protease, an activator of the coagulation system that is activated by DNA and histones, together with plasmin and clotting factors (e.g., thrombin and factor Xa) can generate C3a/C3b and C5a/C5b [32, 36]. Together, this might explain why patients with knee injury with OC fractures who have major cell osteocyte and chondrocyte death and intra-articular bleeding also have increased complement factor activation of C4d and sTCC compared with patients without such fractures, as shown in our present study.

Previously, we showed that patients with recent knee injury have signs of cartilage degradation, as reflected by an increased release into synovial fluid over several weeks of proteolytic products of aggrecan, type II collagen and COMP [14–17, 19, 23, 37, 38]. Many of the ECM proteins and their fragments, such as aggrecan, chondroadherin, fibromodulin and osteoadherin, activate the classical complement pathway [6, 7, 27, 39], while others, such as COMP and type II collagen, activate the alternative pathway [5, 40]. However, several ECM proteins also inhibit complement: Proline/arginine-rich end leucine-rich repeat protein (PRELP) and the NC4 domain of type IX collagen directly inhibit formation of MAC [41, 42]; biglycan, decorin and COMP inhibit the classical pathway via binding of C1q [5, 43]; and PRELP inhibits the alternative pathway [41]. The ECM proteins and their fragments thus seem to have dual effects on complement, either activating or inhibiting it. This duality could in part explain why in the synovial fluid from the recent injury group no correlations were found between the cartilage protein fragments and the components of the different complement pathways.

Compared with individuals with healthy knees, TNF-α concentrations in synovial fluid are increased in patients with OA and also in patients with recent (weeks) as well as old (years) knee injuries [14, 38, 44–46]. Interestingly, in this study, the strongest correlation for the recent injury group samples (0–83 days after injury) was between synovial fluid concentrations of C4d and TNF-α (*r*_s_ = 0.547), and the levels of C4d were also found to be elevated many years after injury. This resembles what was found for patients with ACL injury, who had elevated synovial fluid levels of TNF-α 5 years after injury [38].

In injured knee joints, proinflammatory cytokines are produced by chondrocytes and synoviocytes and by infiltrated leucocytes, resulting in the production and activation of matrix metalloproteases (MMPs) and aggrecanases [11, 47, 48]. As a parallel route, complement components such as sublytic concentrations of MAC stimulate chondrocytes to produce MMPs and aggrecanases [27], and complement anaphylatoxins (C3a and C5a) stimulate granulocytes, macrophages, and B and T cells to produce proinflammatory cytokines [3]. Further, it has been shown that C1s (a protease of the classical pathway) can activate proMMP-9, and C1s can also cleave types I, II and IV collagens [49, 50]. This indicates that there are several possible routes for the induction of extracellular proteases in these patients.

In patients with a recent knee injury (0–5 weeks), the release into the synovial fluid of proinflammatory cytokines IL-1β, IL-6 and TNF-α precedes the release of cartilage degradation products of aggrecan, COMP and type II collagen [14, 19, 23, 38]. In the same patients in the present study, we found no correlation between the complement factors C4d, C3bBbP and sTCC and cartilage degradation products of aggrecan, type II collagen and COMP; however, the complement factor concentrations correlated positively with proinflammatory cytokine levels of IL-1β, IL-6 and TNF-α. This supports the concept that complement together with coagulation represents an alternative route to activation of cytokine pathways, and together these routes drive the catabolic process seen as cartilage degradation after joint injury and most likely later in OA.

This study has certain limitations. First, the lack of dilution linearity for the C3bBbP and sTCC assays using synovial fluid samples complicated the assessments, and only relative comparison could be made by using the same dilution (1:20) for all samples. Second, because the subjects in the OA, RA and PPA groups were older than the subjects in the reference group, we cannot exclude the possibility that the increased levels of the complement factors found in these patient groups could be due partly to differences in age. Third, the knee injury group was cross-sectional, so the design precludes drawing firm conclusions of trends in time because no repeated sampling was done within individual patients. Hence, our suggestions regarding differences in complement activation over time need to be confirmed in longitudinal studies. Finally, we measured only the soluble components of C4d, C3bBbP and sTCC found in synovial fluid, and these also ought to be analysed as cell-bound (e.g., chondrocytes, synoviocytes) components for the different diagnostic groups.

## Conclusions

We show that alternative and classical and/or lectin complement pathways are activated in the synovial fluid of subjects with knee injury and in patients with OA. After an injury to the knee, the complement factors C4d, C3bBbP and sTCC are instantly activated and are associated with proinflammatory cytokines, while C4d and sTCC are associated with OC fractures.

This and previous reports have suggested that complement is activated in OA [27]. Knee injury is a well-known and strong risk factor for rapid knee OA development, but the driving mechanisms are not well understood. The sudden activation of complement after knee injury found in this study, especially among those with more severe trauma, most likely represents one of several important pathways involved in the onset of this complex disease. Therefore, more clinical studies using longitudinal cohorts are needed to elucidate these pathological pathways to determine if or when to use complement inhibitors as a treatment strategy for OA.

## Additional files

Additional file 1: Table S1. Concentrations of C4d, C3bBbP and sTCC in synovial fluid. (DOCX 37 kb)
Additional file 2: Table S2. Technical performance of the C4d, C3bBbP and sTCC immunoassays using synovial fluid. (DOCX 33 kb)
Additional file 3: Table S3. The effect of age, sex and time after injury on the concentrations of C4d, C3bBbP and sTCC in synovial fluid. (DOCX 31 kb)

## Abbreviations

ACL: Anterior cruciate ligament
ANCOVA: Analysis of covariance
BML: Bone marrow lesion
CAU: Complement activation units
COMP: Cartilage oligomeric matrix protein
CV: Coefficient of variation
ECM: Extracellular matrix
ELISA: Enzyme-linked immunosorbent assay
Ig: Immunoglobulin
IL: Interleukin
LLOD: Lower limit of detection
MAC: Membrane attack complex
MASP: Mannose-binding lectin-associated serine protease
MBL: Mannose-binding lectin
MMP: Matrix metalloprotease
MRI: Magnetic resonance imaging
NA: Not applicable
OA: Osteoarthritis
OC: Osteochondral
P: Properdin
PCL: Posterior cruciate ligament, PPA, Pyrophosphate arthritis
PRELP: Proline/arginine-rich end leucine-rich repeat protein
PRM: Pattern recognition molecule
RA: Rheumatoid arthritis
sGAG: Sulfated glycosaminoglycan
SPARC: Secreted protein acidic and rich in cysteine
sTCC: Soluble terminal complement complex
TNF: Tumour necrosis factor
ULOD: Upper limit of detection
ARGS: ARGS-aggrecan, C2C, type II collagen epitope C2C
