# Supplementary material for: The complement system is activated in synovial fluid from subjects with knee injury and from patients with osteoarthritis
Source: Arthritis Res Ther. 2016 Oct 6;18:223. doi: 10.1186/s13075-016-1123-x (PMC5052889; doi:10.1186/s13075-016-1123-x)
Supplement: Additional file 1: Table S1. — Concentrations of C4d, C3bBbP and sTCC in synovial fluid. (DOCX 37 kb) [file 13075_2016_1123_MOESM1_ESM.docx]

**Table S1** Concentrations of C4d, C3bBbP and sTCC in synovial fluid

| **C4d** |  |  |  |  |  |  |
| --- | --- | --- | --- | --- | --- | --- |
|  | Groups | Conc. (CAU) | Norm | P value | n | n < LLOD (%) |
|  | Reference | 2.81 (1.69, 4.04) | 1 | - | 23 | 1 (4) |
|  | Osteoarthritis | 11.23 (6.00, 22.51 | 4.0 | **<0.001** | 24 | 1 (4) |
|  | Rheumatoid arthritis | 93.99 (31.56, 185.77) | 33.5 | **<0.001** | 32 | 1 (3) |
|  | Pyrophosphate arthritis | 32.66 (13.92, 60.05) | 11.6 | **<0.001** | 25 | 1 (4) |
|  | Knee injury | 18.69 (6.41, 44.73) | 6.7 | **<0.001** | 294 | 9 (3) |
|  | Recent injury | 25.11 (11.47, 51.66) | 8.9 | **<0.001** | 219 | 4 (2) |
|  | *0 days* | *34.69 (19.27, 69.11)* | *12.4* | ***<0.001*** | *35* | *1 (3)* |
|  | *1 day* | *39.73 (22.00, 84.7)* | *14.1* | ***<0.001*** | *39* | *1 (3)* |
|  | *2-3 days* | *32.37 (16.32, 47.06)* | *11.5* | ***<0.001*** | *31* | *1 (3)* |
|  | *4-7 days* | *31.41 (16.57, 51.81)* | *11.2* | ***<0.001*** | *46* | *0 (0)* |
|  | *8-22 days* | *14.31 (6.31, 41.85)* | *5.1* | ***<0.001*** | *44* | *1 (2)* |
|  | *23-83 days* | *8.15 (4.61, 19.33)* | *2.9* | ***<0.001*** | *24* | *0 (0)* |
|  | Old injury | 5.09 (2.59, 12.25) | 1.8 | **0.009** | 75 | 5 (7) |
|  | *1-3 years* | *4.48 (2.82, 10.95)* | *1.6* | ***0.006*** | *35* | *2 (6)* |
|  | *3-36.9 years* | *5.20 (1.98, 12.62)* | *1.9* | ***0.048*** | *40* | *3 (8)* |
| **C3bBbP** |  |  |  |  |  |  |
|  | Groups | Conc. (CAU) | Norm | P value | n | n < LLOD (%) |
|  | Reference | 22.02 (7.93, 65.83) | 1 | - | 23 | 3 (13) |
|  | Osteoarthritis | 71.40 (37.07, 101.21) | 3.2 | **0.008** | 24 | 1 (4) |
|  | Rheumatoid arthritis | 110.54 (88.47, 218.75) | 5.0 | **<0.001** | 32 | 0 (0) |
|  | Pyrophosphate arthritis | 40.40 (25.41, 75.67) | 1.8 | **0.044** | 25 | 0 (0) |
|  | Knee injury | 101.62 (45.15, 217.46) | 4.6 | **<0.001** | 294 | 6 (2) |
|  | Recent injury | 144.22 (72.52, 250.93) | 6.6 | **<0.001** | 219 | 1 (0.5) |
|  | *0 days* | *221.12 (118.06, 361.60)* | *10.0* | ***<0.001*** | *35* | *0 (0)* |
|  | *1 day* | *208.69 (134.34, 359.28)* | *9.5* | ***<0.001*** | *39* | *0 (0)* |
|  | *2-3 days* | *152.28 (89.78, 234.68)* | *6.9* | ***<0.001*** | *31* | *0 (0)* |
|  | *4-7 days* | *145.73 (78.72, 243.03)* | *6.6* | ***<0.001*** | *46* | *0 (0)* |
|  | *8-22 days* | *91.83 (37.41, 178.69)* | *4.2* | ***<0.001*** | *44* | *0 (0)* |
|  | *23-83 days* | *61.75 (33.31, 89.16)* | *2.8* | ***0.007*** | *24* | *1 (4)* |
|  | Old injury | 36.47 (18.75, 73.31) | 1.7 | 0.110 | 75 | 5 (7) |
|  | *1-3 years* | *29.58 (14.01, 58.64)* | *1.3* | *0.230* | *35* | *1 (3)* |
|  | *3-36.9 years* | *38.17 (21.41, 87.31)* | *1.7* | *0.101* | *40* | *4 (10)* |
| **sTCC** |  |  |  |  |  |  |
|  | Groups | Conc. (CAU) | Norm | P value | n | n < LLOD (%) |
|  | Reference | 1.97 (1.19, 5.45) | 1 | - | 23 | 4 (17) |
|  | Osteoarthritis | 7.79 (4.19, 11.33) | 4.0 | **<0.001** | 24 | 0 (0) |
|  | Rheumatoid arthritis | 24.35 (10.68, 40.46) | 12.4 | **<0.001** | 32 | 0 (0) |
|  | Pyrophosphate arthritis | 22.09 (17.78, 33.29) | 11.2 | **<0.001** | 25 | 0 (0) |
|  | Knee injury | 8.48 (4.68, 13.54) | 4.3 | **<0.001** | 294 | 4 (1) |
|  | Recent injury | 10.12 (6.78, 15.14) | 5.2 | **<0.001** | 219 | 1 (0.5) |
|  | *0 days* | *9.65 (6.60, 16.17)* | *4.9* | ***<0.001*** | *35* | *0 (0)* |
|  | *1 day* | *11.71 (7.86, 19.56)* | *6.0* | ***<0.001*** | *39* | *0 (0)* |
|  | *2-3 days* | *15.59 (10.15, 20.18)* | *7.9* | ***<0.001*** | *31* | *0 (0)* |
|  | *4-7 days* | *11.07 (7.66, 14.22)* | *5.6* | ***<0.001*** | *46* | *0 (0)* |
|  | *8-22 days* | *8.66 (5.68, 11.81)* | *4.4* | ***<0.001*** | *44* | *1 (2)* |
|  | *23-83 days* | *5.44 (4.07, 9.13)* | *2.8* | ***0.001*** | *24* | *1 (4)* |
|  | Old injury | 3.33 (1.58, 7.46) | 1.7 | 0.082 | 75 | 3 (4) |
|  | *1-3 years* | *3.21 (1.48, 7.63)* | *1.6* | *0.125* | *35* | *2 (6)* |
|  | *3-36.9 years* | *3.71 (1.62, 7.44)* | *1.9* | *0.113* | *40* | *1 (3)* |

Concentrations (conc.) in synovial fluid expressed as median values (25^th^, 75^th^ percentiles) were measured in different diagnostic groups (Table 1). Median values are normalized (Norm) against the reference group. P values, Mann-Whitney U tests of patients versus reference group. Significances (p < 0.05) are marked bolded. n = total amount of samples analyzed. n < LLOD = amount of the analyzed samples that were below lower limit of detection. CAU = complement activation unit.
