# Supplementary material for: The complement system is activated in synovial fluid from subjects with knee injury and from patients with osteoarthritis
Source: Arthritis Res Ther. 2016 Oct 6;18:223. doi: 10.1186/s13075-016-1123-x (PMC5052889; doi:10.1186/s13075-016-1123-x)
Supplement: Additional file 2: Table S2. — Technical performance of the C4d, C3bBbP and sTCC immunoassays using synovial fluid. (DOCX 33 kb) [file 13075_2016_1123_MOESM2_ESM.docx]

**Table S2** Technical performance of the C4d, C3bBbP and sTCC immunoassays using synovial fluid

| Analyte |  |  | C4d | C3bBbP | sTCC |
| --- | --- | --- | --- | --- | --- |
| Detection, CAU |  |  |  |  |  |
|  |  | LLOD | 0.05 | 0.05 | 0.02 |
|  |  | ULOD | 25 | 50 | 10 |
| Recovery, mean (range) % |  |  |  |  |  |
|  | Dilution | Ratio |  |  |  |
|  |  |  | n=12 | n=6 | n=12 |
|  |  | 1:5 | 97 (59-119) | 54 (42-76) | 60 (49-83) |
|  |  | 1:10 | 101 (73-134) | 82 (67-90) | 84 (77-98) |
|  |  | 1:20 | 98 (81-122) | 114 (103-119) | 115 (103-120) |
|  |  | 1:40 | 104 (78-146) | 150 (135-176) | 141 (105-163) |
|  | Spiking | CAU |  |  |  |
|  |  |  | n=4 or 6 | n=4 | n=2, 4 or 5 |
|  | High conc. | 2.50 | 79 (75-86) | 96 (81-122) | 68 (68-68) |
|  | Middle conc. | 0.63 | Nd | 107 (89-147) | 77 (62-94) |
|  | Low conc.^1^ | 0.13, 0.31 | 99 (81-125) | Nd | 77 (71-84) |
|  | Freeze-thaw | Cycle |  |  |  |
|  |  |  | n=6 | n=6 | n=4 |
|  |  | 3^rd^ | 109 (91-147) | 111 (88-140) | 118 (96-140) |
|  |  | 7^th^ | 106 (84-140) | 124 (74-177) | 104 (93-113) |
|  |  | 15^th^ | 113 (76-154) | 109 (76-153) | 98 (93-103) |
| CV, mean (range) % |  |  |  |  |  |
|  |  |  | n=4 | n=4 | n=4 |
|  |  | Intra | 9.7 (7.2-14.5) | 10.2 (6.2-12.2) | 8.8 (6.8-12.5) |
|  |  | Inter | 15.9 (10.8-20.9) | 22.7 (15.6-32.5) | 23.4 (20.6-26.0) |

To calculate dilution linearity randomly selected synovial fluid samples were prepared in different dilutions and analyzed. Results are expressed as % recovery: 100 x [(concentration at a specific dilution) divided by (original concentration divided by the dilution times)]. Spiking recovery was calculated from randomly selected synovial fluid samples which were spiked using standards. Spiking recovery is expressed in % as recovery: 100 x [(concentration of a sample spiked with a specific amount of standard) divided by (the individual concentration of sample plus the individual concentration from standard)]. To analyze freeze-thawing effects on C4d, C3bBbP and sTCC concentrations randomly selected synovial fluid samples were freeze/thawed fifteen cycles. Recovery after freeze-thaw cycles is expressed in relation to what was obtained the first time the sample was thawed. Intra and inter C4d, C3bBbP and sTCC assay coefficient of variations (CV) was calculated using randomly selected synovial fluid samples loading 4 repeats (intra) on a single plate or loading duplicates on 5-6 plates (inter). Lower limit of detection (LLOD) and upper limit of detection (ULOD) for the C4d, C3bBbP and sTCC assays were estimated. CAU = complement activation unit. Nd = not determined. 1) Spiking was done with CAU = 0.13 (C4d) or with CAU = 0.31 (sTCC).
