# Supplementary material for: The complement system is activated in synovial fluid from subjects with knee injury and from patients with osteoarthritis
Source: Arthritis Res Ther. 2016 Oct 6;18:223. doi: 10.1186/s13075-016-1123-x (PMC5052889; doi:10.1186/s13075-016-1123-x)
Supplement: Additional file 3: Table S3. — The effect of age, sex and time after injury on the concentrations of C4d, C3bBbP and sTCC in synovial fluid. (DOCX 31 kb) [file 13075_2016_1123_MOESM3_ESM.docx]

**Table S3** The effect of age, gender and time after injury on the concentrations of C4d, C3bBbP and sTCC in synovial fluid

|  | Reference,  n=23 | | | | Osteoarthritis,  n=24 | | | | | Rheumatoid arthritis, n=32 | | | Pyrophosphate arthritis,  n=25 | | | | |  |
| --- | --- | --- | --- | --- | --- | --- | --- | --- | --- | --- | --- | --- | --- | --- | --- | --- | --- | --- |
|  | Gender | | Age | | Gender | | Age | | | Gender | Age | | Gender | | Age | | |  |
| C4d | 0.403 | | **0.426 (0.043)** | | 0.585 | | 0.257 (0.225) | | | 0.920 | 0.146 (0.427) | | 0.807 | | 0.386 (0.057) | | |  |
| C3bBbP | 1.000 | | 0.117 (0.596) | | 0.312 | | -0.101 (0.638) | | | 0.096 | 0.096 (0.608) | | 0.807 | | 0.083 (0.858) | | |  |
| sTCC | 0.991 | | 0.180 (0.410) | | 0.056 | | **0.407 (0.048)** | | | 0.326 | 0.257 (0.156) | | 0.531 | | **0.484 (0.014)** | | |  |
|  | | Knee injury,  n=294 | | | | | | Recent injury,  n=219 | | | | | | Old injury,  n=75 | | | | |
|  | | Gender | | Age | | Time | | Gender | Age | | | Time | | Gender | | Age | Time | |
| C4d | | 0.743 | | **-0.251 (<0.001)** | | **-0.519 (<0.001)** | | 0.679 | **-0.246 (<0.001)** | | | **-0.319 (<0.001)** | | 0.593 | | 0.151 (0.196) | 0.010 (0.929) | |
| C3bBbP | | 1.000 | | **-0.362 (<0.001)** | | **-0.571 (<0.001)** | | 0.440 | **-0.308 (<0.001)** | | | **-0.407 (<0.001)** | | 0.719 | | -0.216 (0.063) | 0.090 (0.443) | |
| sTCC | | 0.991 | | **-0.149 (0.011)** | | **-0.503 (<0.001)** | | 0.370 | -0.086 (0.205) | | | **-0.301 (<0.001)** | | 0.198 | | 0.083 (0.479) | 0.066 (0.571) | |

Age and gender distributions and concentrations of C4d, C3bBbP and sTCC in synovial fluid are presented for the different subject groups in Table 1 and Table S2. Differences, using Mann-Whitney U tests, in biomarker concentrations between men and women (gender) are shown with p-values. Correlation, using Spearman rho (r_S_), between biomarker concentrations and age, and between biomarker concentrations and time after injury are shown as r_S_-values with p-values in brackets. Significances (p < 0.05) are marked bolded.
